# The Guild of Social Workers

**Published:** 1917-04-21

**Authors:** 


### THE BEDFORD COUNTY HOSPITAL.

### THE GUILD OF SOCIAL WORKERS.

### A Successful Experiment.

The annual meeting of the Hospital Guild for Bedfordshire was held on Friday, April 13, in the Hall of the Bedford County Hospital, where the first landing of the staircase served as a platform. Lady Ampthill presided, supported by Mrs. Whitbread, Mrs. Gurney, Sir Henry Burdett, K.C.B., Mr. H. C. Williams, Mr. J. Arnold Whitchurch, Mr. W. Gifford Nash (Vice-President), Mr. H. S. Deacon, Mr. F. Diemer. Among others present were Major-General and Mrs. Layard, Colonel Hay Shaw, Major Carpenter, Miss Whitchurch, Mrs. Eleanor Paine, Mr. Gostick, the Rev. G. C. Graham, General Inglefield, Mrs. Josselyn, Mr. J. Adams, Mr. Beauchamp Wadmore, and others.

Lady Ampthill said they deeply deplored the loss of Lady Isabella Whitbread, who had been President of the Guild from its commencement, and though reasons of health had in late years prevented her taking an active share, she always took a great interest in the working. The fact that Mrs. Whitbread had come to propose the adoption of the report was sufficient evidence that the new generation would take the same interest in the hospital as had Lady Isabella. They were also fortunate in securing the support of Sir Henry Burdett, than whom no one knew more about hospitals and their management. His works on the subject were classics, and his presence was a great encouragement; they were hoping to hear from him much advice on how to expand the usefulness of this work. In spite of a terrible war they had been able to go on quietly with the work, and though it had not been possible to organise entertainments all over the county, as in happier times, there was one that produced wonderful results at Great Barford on Easter Monday. Wishing to do something for the hospital of which Mr. Whitchurch was chairman, the village had a sale in the afternoon, a concert in the evening, finished up with a dance, and sent £86 3s. 6d. to the hospital, besides quantities of eggs and potatoes. The house-to-house collections had increased wonderfully, and the good work of Mrs. Seys Phillips for the Needlework Guild was much appreciated. They regretted that Colonel Hay Shaw was resigning the chairmanship of the After Care of Patients Committee, and the Guild owed a debt of gratitude to him for his loyal support. The County Hospital was foremost in every good work, and they were all very proud of it.

Mrs. Whitbread moved the adoption of the report, and said no one, after reading the record of the splendid work of the Guild, could believe that the hospital would be starved for want of effort on its behalf. This county loved its hospital, and the collectors had gone on collecting year after year for the hospital. No one liked asking for money, but the collectors knew that the money must be got, and last year they collected £894 11s. 4d. The war had taught them that people could not gauge the efforts they were capable of. No one would have thought it possible to raise such an army in this country, or that the women of this country could make munitions, but each succeeding year and month exceeded the past and reached new standards, and she thought that even the present record would be eclipsed. The number of in-patients treated in the hospital was 891; the average cost per bed occupied was £83 11s. 3d.; and a debt of £746 would be wiped out.

Mrs. Gurney seconded with great pleasure, and spoke of two branches of the work which fulfilled one object. One was increasing the resources of the hospital by doing supplemental charitable work, and the other was the activity of the Needlework Guild in supplying garments for the use of the patients in the wards. Their ideals were growing, and happily they had willing workers to see that these ideals were realised. It was difficult to imagine how the hospital was carried on in days gone by, when there was no Needlework Guild, and many of the very poor had no change of linen or bed garments. They were told of nurses who faced the difficulty by buying garments with their own money, and in their much-needed hours of rest they made the garments for the worst cases. Those days were happily over, and the committee had a good stock of garments in hand. Owing to better conditions the demand for them was not so great, and for some years they had appealed for household linen, blankets and sheets, or, better still, for money to buy them with. Since the first exhibition in 1908 the number of things had steadily increased, and it spoke volumes for the enthusiasm of the workers that the articles had grown in number from 300 in 1908 to 942 in 1916. The hospital had been supplied, the funds helped, and great relief and comfort afforded to the nursing staff. The patients must also admire and appreciate the nice things provided for them. It was difficult to exaggerate the importance of the work. The organisation was surely as perfect as possible. While no case could be overlooked, none was helped with money unless there was real need. If possible, the hospital authorities communicated with the President some days before the discharge of patients, giving particulars of the assistance needed. Inquiries could be made as to means and circumstances, and there was no delay in giving necessary help. If nourishment was needed, the case was handed to the Ladies' Committee, members visited and helped, and saw that the food recommended was provided. In giving other help, the committee co-operate with the Bedford C.O.S. Many patients were sent to sanatoria, convalescent homes, or for change in the country, and helped with medical and surgical appliances. Over 100 cases had been sent to these places, and 2,904 dinners had been given, not counting the thousands of puddings, and 6,000 pints of milk. All this had been necessary for the complete recovery of the patient. The moral and spiritual good of this work could not be gauged. They knew something of the weariness and distress of serious illness, and the good done for their poor neighbours in times of difficulty could not be calculated.

The report was adopted, and a vote of thanks to Lady Ampthill for presiding was passed with acclamation.

